# Origin-Dependent Molecular Ordering in Gelatin and Its Impact on Electrospun Nanofiber

**DOI:** 10.3390/polym17162219

**Published:** 2025-08-14

**Authors:** Seong Baek Yang, Yu Kyung Lee, Dong-Jun Kwon

**Affiliations:** 1 Research Institute for Green Energy Convergence Technology, Gyeongsang National University, Jinju 52828, Republic of Korea; sbyang@gnu.ac.kr; 2 Department of Materials Science and Convergence Technology, Gyeongsang National University, Jinju 52828, Republic of Korea; lyk8681@naver.com

**Keywords:** electrospinning, nanofiber, gelatin, bovine, porcine, fish

## Abstract

Electrospun nanofibrous mats from bovine, porcine, and fish gelatin were systematically fabricated at varying concentrations (15, 20, 25, and 30 wt.%) to investigate the influence of molecular characteristics on morphology, crystallinity, mechanical properties, thermal behavior, and solubility. Optimal ranges of viscosity (0.08–1.47 Pa·s), surface tension (35–50 mN·m^−1^), and electrical conductivity (0.18–1.42 mS·cm^−1^) were determined to successfully produce homogeneous fibers. Bovine and porcine gelatin, characterized by higher molecular weight and greater proline/hydroxyproline content, exhibited thicker (up to 725 ± 41 nm at 30 wt.%) and less uniform nanofibers due to higher viscosity and surface tension, restricting polymer jet stretching. Conversely, fish gelatin, with lower molecular weight and limited proline/hydroxyproline content, produced significantly thinner (as low as 205 ± 28 nm at 20 wt.%) and more uniform nanofibers. X-ray diffraction analysis revealed distinct crystallinity transitions associated with triple-helix and amorphous structures, dependent on gelatin type and concentration, including the emergence of peaks near 7.9° and 20.1° (2θ) for bovine gelatin. Mechanical tests demonstrated superior tensile strength for bovine gelatin (up to 2.9 MPa at 30 wt.%), balanced properties for porcine gelatin, and exceptional elasticity for fish gelatin. Thermal analysis indicated concentration-dependent shifts in viscoelastic behavior and damping performance. Solubility studies showed rapid dissolution of low-concentration fish gelatin fibers, moderate stability for intermediate-concentration porcine gelatin, and excellent structural retention for high-concentration bovine gelatin. These results demonstrate the potential for tailored gelatin nanofiber design to meet specific functional requirements in biomedical applications.

## 1. Introduction

Recently, nanofiber fabrication technology has gained significant attention across diverse application fields. In particular, electrospinning, which utilizes high voltage to create ultrafine fibers ranging from tens to hundreds of nanometers, offers an extensive specific surface area and high porosity, making it increasingly applicable in medical, environmental, and energy storage sectors [1,2]. Since the early 2000s, advancements such as material diversification, core–shell structures, and multi-axis electrospinning have emerged, and electrospun nanofibers from natural polymers have garnered attention in tissue engineering due to their inherent biocompatibility and biodegradability [3,4]. With the advancement of electrospinning technology, the applications of nanofibers have significantly broadened, ranging from filtration, sensors, and catalyst supports to wound healing, drug delivery, and tissue regeneration. In particular, within biomedical fields, electrospun nanofibers have gained attention as a core technology in tissue engineering and regenerative medicine due to their ability to mimic the nanostructure of the extracellular matrix. Electrospinning performance is highly influenced by solution parameters including viscosity, surface tension, and electrical conductivity. If viscosity is too low, fiber formation becomes challenging, resulting in defects; too-high viscosity restricts solution flow, causing instability. In electrospinning, highly viscous solutions induce several specific instabilities that hinder fiber formation [5,6]. When the viscosity increases excessively, the viscoelastic forces of the polymer solution interfere with the stretching of the polymer jet by the electric field, resulting in irregular jet flow, reduced jet travel distance, and droplet accumulation at the needle tip, which can lead to nozzle clogging and instability of the Taylor cone [7,8,9]. As viscosity increases, the flow of the solution through the syringe becomes restricted, making it difficult to control the flow rate and thereby increasing the likelihood of unstable jet formation and interruption of the continuous electrospinning process. Moreover, in highly viscous solutions, thick polymer strands may form at the needle tip and merely oscillate within the electric field or detach as unstretched polymer masses, leading to fiber formation failure when these aggregates are deposited onto the collector [8,10]. Similarly, inappropriate surface tension can destabilize the spinning process, hindering uniform fiber production, whereas suitable surface tension promotes stable and consistent fiber formation. Surface tension is a critical parameter that governs the success of electrospinning through specific mechanisms [8,11]. When surface tension exceeds the electrostatic stretching force, Rayleigh instability is induced, leading to jet breakage and the formation of bead-on-string structures. In particular, surface tension values above 55 mN/m increase the tendency of liquid jets to retract into spherical droplets, thereby suppressing fiber formation. Conversely, an optimal surface tension range (typically 30–55 mN/m for most polymer systems) achieves a balance with electrostatic forces, enabling stable Taylor cone formation and sustained jet elongation during the whipping instability phase, ultimately resulting in uniform fiber generation [12,13]. However, in our study, successful electrospinning was achieved even at surface tension values slightly exceeding the conventional upper limit (i.e., >55 mN/m), likely due to the compensatory effects of optimized viscosity and electrical conductivity, which collectively stabilized the jet behavior and suppressed droplet formation. In addition, high surface tension can be effectively counterbalanced by applying a higher voltage, a scientifically valid mechanism grounded in Taylor’s critical voltage theory and the principles of electrohydrodynamic force balance [7]. In electrospinning, surface tension and applied voltage are interdependent parameters, and the application of sufficiently high voltage can overcome the limitations imposed by elevated surface tension, thereby enabling stable Taylor cone formation and continuous fiber elongation. In fact, Li et al. demonstrated that electrospinning is possible even under extremely low-voltage conditions (as low as 50 V), which indirectly supports the principle that high surface tension can be overcome by increasing the applied voltage [14]. Their results indicate that as surface tension increases, a higher critical voltage is required, suggesting a direct compensatory relationship between the two parameters. Furthermore, the study by Reneker provided scientific evidence for this mechanism by showing that when sufficient voltage is applied, the electrostatic force can overcome surface tension, enabling the formation of a stable Taylor cone and continuous jet initiation [15]. These prior findings substantiate the theoretical basis for using high voltage to compensate for high surface tension, thereby validating the feasibility of the experimental approach employed in our study. Electrical conductivity affects how efficiently the electric field influences the solution; excessive conductivity can lead to unstable jet behaviors and difficulty in controlling fiber uniformity.

Gelatin, derived from collagen through thermal and acid/alkali treatments, is a natural protein-based polymer suitable for medical applications owing to its excellent biodegradability and biocompatibility [4,16]. However, gelatin nanofibers face challenges including rapid dissolution in aqueous environments, limited mechanical strength and thermal stability, and variations in process parameters depending on the gelatin source [17,18]. To address these issues, this study systematically examines different gelatin sources (bovine, porcine, fish) and concentrations (15, 20, 25, 30 wt.%) to simultaneously optimize solubility, mechanical, and thermal performance, thus effectively balancing the inherent trade-offs among dissolution, strength, and processability. Furthermore, this study aims to clearly define the optimal ranges of critical electrospinning parameters such as viscosity, surface tension, and electrical conductivity, providing precise material control guidelines for gelatin nanofibers. Some limitations, including rapid aqueous dissolution, limited mechanical strength, and inconsistent thermal stability, pose significant barriers to the clinical application and commercialization of gelatin nanofibers; thus, systematic elucidation of the correlation between gelatin’s molecular characteristics and electrospinning process variables is essential to overcome them [16]. While previous studies have mostly focused on a single gelatin type or limited concentration ranges, resulting in a lack of comprehensive design guidelines, this study aims to address these research gaps [19].

Gelatin is primarily categorized into bovine, porcine, and fish types. Bovine gelatin, characterized by high proline and hydroxyproline content (~30 mol%) and medium molecular weight (45–65 kDa), demonstrates superior mechanical strength. Proline and hydroxyproline are amino acids derived from collagen that possess rigid cyclic structures; hydroxyproline additionally contains a hydroxyl group that enhances hydrogen bonding [20,21,22]. These amino acids play a key role in stabilizing the collagen triple helix through intramolecular interactions. In gelatin, although the native triple helix is denatured, the content of proline and hydroxyproline remains a critical factor influencing thermal stability, gel strength, and mechanical performance [23,24]. Porcine gelatin, with its high molecular weight (80–105 kDa), exhibits extensive chain entanglements, facilitating controlled solubility. Fish gelatin, having a lower molecular weight (20–30 kDa) and lower proline/hydroxyproline content (~17 mol%), exhibits weaker triple-helix formation, resulting in increased aqueous solubility [25,26]. Collagen possesses a robust triple-helix structure crucial for stability; however, gelatin, a denatured collagen form, features a disrupted triple-helix structure due to thermal and chemical processing, resulting predominantly in amorphous coil structures. Electrospun gelatin nanofibers generally exhibit an amorphous structure, though some degree of molecular alignment may occur under electric fields. These molecular differences directly influence the gelation temperature, viscoelastic behavior, and aqueous stability of gelatin, serving as critical determinants of the final properties of electrospun nanofibers. Therefore, understanding the intrinsic molecular characteristics of each gelatin type and optimizing these parameters in the electrospinning process are prerequisites for fabricating nanofibers tailored to specific application requirements.

The electrospinning process generally involves jet initiation, elongation, and fiber collection, which are strongly influenced by key solution properties such as viscosity, surface tension, and electrical conductivity. In this study, the effects of gelatin type and concentration on these solution parameters were systematically analyzed in relation to fiber formation, mechanical strength, and solubility, aiming to provide practical design guidelines for biomedical nanofiber applications. These key parameters were precisely investigated to establish reproducible electrospinning protocols optimized for each gelatin type [27,28].

The objective of this research is to quantitatively elucidate the effects of gelatin sources and concentration combinations on the structural, mechanical, thermal, and aqueous solubility properties of electrospun nanofibers and provide tailored design guidelines for specific application purposes. Specifically, morphological analysis using SEM, molecular structural identification using XRD and FT-IR, mechanical and thermal performance evaluation using DMA, and aqueous solubility testing were conducted. These analyses aim to propose optimal gelatin compositions for targeted applications such as wound dressings, drug delivery systems, and temporary tissue scaffolds, and establish a three-dimensional “source–concentration–process” design space for practical utilization of gelatin nanofibers [19]. Previous research typically focused on individual gelatin types or limited concentration ranges, which has hindered the development of comprehensive design guidelines [29].

## 2. Materials and Methods

### 2.1. Materials

The materials that were used in this study are gelatins purchased from Sigma-Aldrich (bovine gelatin type B, G9391; porcine gelatin type A, G1890; fish gelatin, G7041, St. Louis, MI, USA). These gelatin types exhibit distinct amino acid compositions, with mammalian gelatins (bovine and porcine) containing higher amino acid content (22.91% and 23.7%, respectively) compared to fish gelatin (19.16–20.86%). The amino acid profiles and structural differences between these gelatin sources have been well-documented in the literature [30,31]. To prepare a solution for electrospinning, as a solvent, we used acetic acid (99.5%, CAS number: 64-19-7, Samchun, Seoul, Republic of Korea). Distilled water was used without further purification.

### 2.2. Electrospinning for Preparing Gelatin Nanofibrous Mats

Gelatin solutions were prepared at concentrations of 15, 20, 25, and 30 wt.% by mixing gelatin with a solvent mixture containing 7.5 mL acetic acid and 2.5 mL distilled water. The mixtures were stirred at 200 rpm for 1 h to achieve homogeneity. Electrospinning was performed using a 10 mL plastic syringe equipped with a 20 G needle. The distance between the needle tip and the aluminum foil-wrapped collector was maintained at 100 mm. The solution was electrospun at a constant flow rate of 0.1 mL/h, an applied voltage of 20 kV, and an ambient temperature of 25 °C to fabricate nanofibrous gelatin. The electrospun gelatin nanofibers were stored in a polypropylene zipper bag for subsequent experiments.

### 2.3. Characteristics of the Prepared Gelatin Nanofibrous Mats

#### 2.3.1. Solution Properties

The viscosity of the gelatin solutions was measured using a viscometer (DV-II, Brookfield, MA, USA) at a constant temperature of 25 °C. The surface tension of gelatin solutions was determined using a surface tension analyzer (DST-30, SEO, Seoul, Republic of Korea). Electrical conductivity of the gelatin solutions was measured using data acquisition equipment (34924A, Agliant, Santa Clara, CA, USA).

#### 2.3.2. Morphological Properties

The electrospun gelatin nanofibrous webs were examined for their morphology using a field-emission scanning electron microscope (FE-SEM, JSM-7600, JEOL, Tokyo, Japan). Small pieces of nanofibrous specimens were fixed onto a stub using carbon tape and coated with platinum using an Auto Fine Coater (JFC-1600, JEOL, Tokyo, Japan) to enhance the electrical conductivity during analysis. The coated samples were then observed under high vacuum using FE-SEM. The diameter of nanofibers was determined by measuring 50 randomly selected nanofibers using Image J (ver. 1.46j, National Institutes of Health, Bethesda, MD, USA) software. The nanofiber’s diameter was measured based on We have added the version information as “ImageJ (version 1.46j, National Institutes of Health, Bethesda, MD, USA).”the scale bar on the FE-SEM micrograph and converted into a number.

#### 2.3.3. Chemical Structure Analysis

A Fourier-transform infrared spectrometer (FTIR, VERTEX-70, Bruker, Billerica, MA, USA) was used to assess the functional groups of the membranes; the spectra were collected over the wavenumber range of 600–4000 cm^−1^ with 16 scans. X-ray diffraction (XRD, D/Max–2500, Rigaku, Tokyo, Japan) was used to analyze the structural characteristics and crystallinity of the electrospun gelatin nanofibers. The XRD patterns were examined to identify changes in molecular organization and to assess the presence of amorphous or semi-crystalline features associated with gelatin structure.

#### 2.3.4. Mechanical Performance

Dynamic mechanical properties of gelatin nanofibrous mats were analyzed using a dynamic mechanical analyzer (DMA, Discovery DMA850, TA instrument, New Castle, DE, USA). DMA tests were conducted at a heating rate of 5 °C/min under a N_2_ atmosphere, within the temperature range of −50 to 200 °C. The nanofibrous mats were prepared by cutting them into dimensions of 50 mm × 10 mm. All samples had a uniform thickness of 0.1 mm, and tensile tests were conducted using a universal tester machine (AG-100kNX plus, Shimadzu Co., Ltd., Kyoto, Japan) at a temperature of 25 °C.

#### 2.3.5. Aqueous Stability

To investigate the dissolution behavior of the nanofibrous mats in cold water, snapshots were captured at an initial timepoint and 30 s using a USB microscope (AM3113, AnMo Electronics Corporation, Taipei, Taiwan).

## 3. Results and Discussion

As summarized in Table 1, the average fiber diameters varied significantly according to gelatin type and concentration, reflecting differences in solution properties under identical electrospinning conditions. Generally, successful electrospinning requires solution parameters—viscosity, surface tension, and electrical conductivity—to fall within specific ranges that enable stable jet elongation and solidification. According to Niehues et al., gelatin solutions with viscosities in the range of 300–700 mPa·s tend to yield uniform nanofibrous mats [32]. While this range has been previously proposed as optimal, our results indicate that the electrospinnability of gelatin solutions is not strictly confined to this range. For instance, fish gelatin formed uniform fibers even below 300 mPa·s, while bovine gelatin required higher viscosities (>1000 mPa·s) to achieve continuous jet elongation. These findings suggest that the optimal viscosity range for electrospinning is gelatin-type-dependent and should be considered in conjunction with other parameters such as surface tension and electrical conductivity.

**Figure 1:**
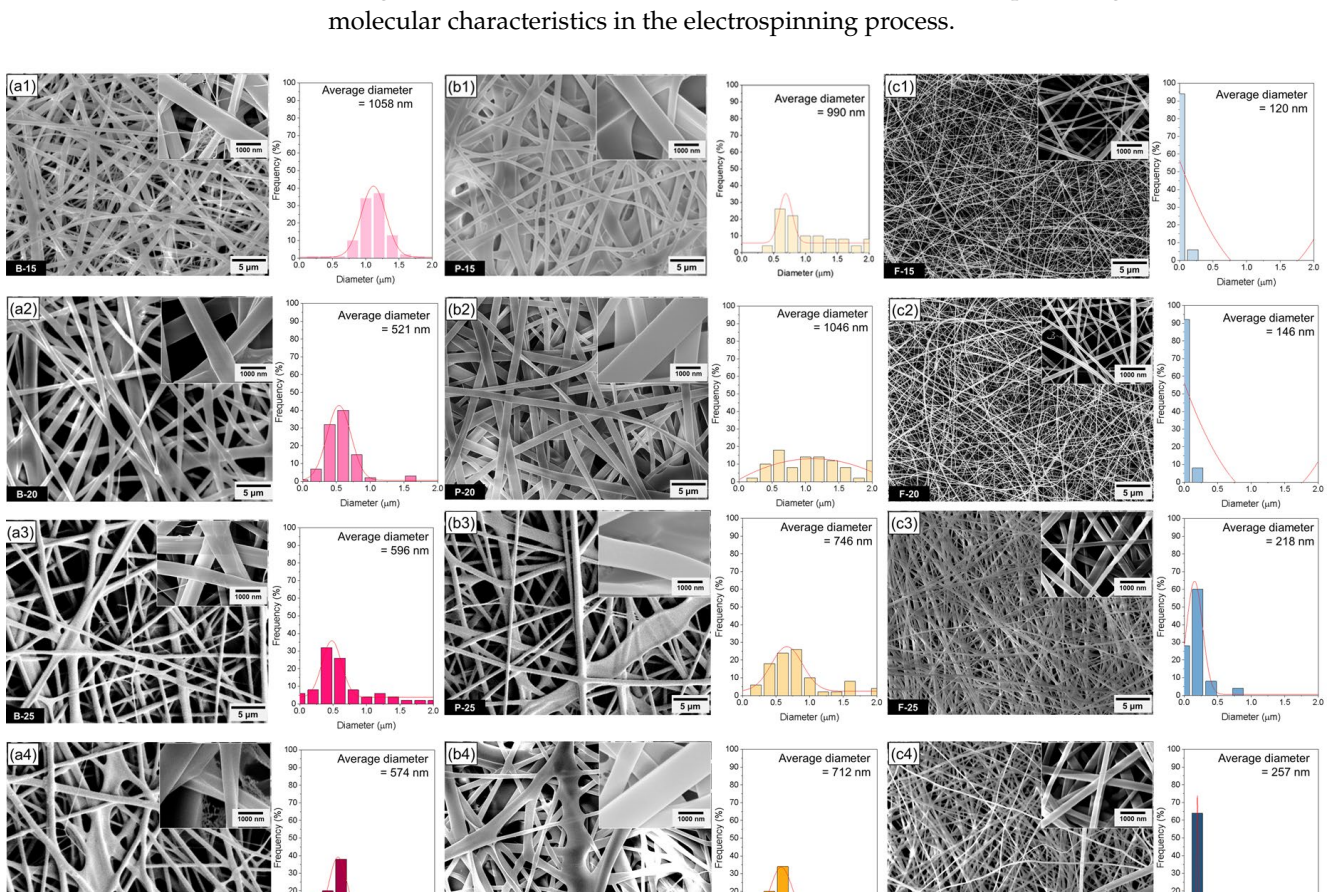
FE-SEM images and diameter distribution including average diameter of electrospun gelatin nanofibrous mats from bovine (**a1**–**a4**), porcine (**b1**–**b4**), and fish gelatin (**c1**–**c4**) at 15, 20, 25, and 30 wt.%.

In this study, bovine and porcine gelatin solutions exhibited markedly higher viscosity and surface tension than fish gelatin solutions, resulting in thicker and more irregular fibers, especially at 15–20 wt.%. Specifically, the viscosity of bovine gelatin increased from 1400 to 7400 mPa·s and that of porcine gelatin from 2000 to 22,126 mPa·s as the concentration increased from 15 to 30 wt.%. In contrast, fish gelatin showed a broader viscosity range, increasing from 400 to 23,552 mPa·s, reflecting its distinct molecular weight distribution and polymer chain dynamics. Although increased viscosity typically leads to thicker fibers due to limited jet stretching, jet instability phenomena—such as Rayleigh–Taylor breakup and whipping-induced jet fragmentation—were observed at higher concentrations (25–30 wt.%), particularly in porcine gelatin. These instabilities led to the unexpected formation of thinner fibers, despite the higher solution viscosity, as partial jet collapse during the whipping stage created localized jet thinning. Fish gelatin solutions, on the other hand, displayed lower viscosity and surface tension, especially at 15–20 wt.%, aligning closely with optimal electrospinnability conditions. Additionally, fish gelatin showed relatively higher electrical conductivity—for example, 1780 μS/cm at 25 wt.%, compared to 1689 μS/cm and 1461 μS/cm for bovine and porcine gelatin, respectively. This enhanced conductivity intensified the electrostatic stretching force on the jet, promoting fiber thinning and uniform elongation. As a result of these favorable solution properties, fish gelatin consistently produced thin and uniform cylindrical nanofibers across all tested concentrations, with diameters approximately one-third to one-half of those obtained from bovine and porcine gelatin. These observations underscore the synergistic effect of viscosity, surface tension, and conductivity in determining fiber morphology, and highlight the critical role of molecular characteristics—such as molecular weight and amino acid composition—in governing electrospinning performance. Optimal conditions for surface tension and electrical conductivity have also been reported, but these parameters are not always strictly considered from the viewpoint of electrospinning apparatus conditions [8]. In this study, we have confirmed experimentally the electrospinnable ranges of surface tension and conductivity under fixed electrospinning conditions (Table 1). As shown in Figure 1, nanofiber diameters significantly varied according to gelatin types due to differences in solution properties under identical processing conditions. Specifically, bovine and porcine gelatin exhibited considerably higher viscosity and surface tension compared to fish gelatin solutions. While higher viscosity generally results in thicker fibers due to restricted jet stretching, at certain higher concentrations (20 wt.%), local jet instability and partial jet collapse phenomena unexpectedly produced thinner nanofibers. Higher-viscosity and -surface-tension conditions during whipping instability, prior to solidification, allowed partial jet fragmentation and led to variations in fiber morphology. The formation of thicker nanofibers can be attributed to insufficient stretching of the polymer jet during the whipping instability stage, prior to solidification, allowing the jet to reach the collector with limited elongation [33,34]. Therefore, in porcine gelatin solutions with concentrations of 25–30 wt.%, the surface tension exceeds the optimal range for electrospinning, leading to the occurrence of localized Rayleigh–Taylor and whipping instabilities. While it is generally known that an increase in polymer concentration results in thicker fiber diameters, recent studies have reported a fiber diameter refinement mechanism induced by instabilities under high-surface-tension conditions. López-Herrera et al. identified that Rayleigh–Taylor breakup occurs during the whipping stage [35], and Jemma R. P. Forgie et al. observed bead-on-string structures along with a decrease in fiber diameter under 25–30 wt.% conditions [36]. Abdallah Refate et al. also reported that higher surface tension shifts the onset of whipping upstream, thereby inducing jet splitting and contraction [37]. Taken together, these findings suggest that in this study, as the gelatin concentration increases, the polymer jet generally maintains a thicker form upon reaching the collector. However, due to jet breakup and instability during its transit, some refined fibers are simultaneously formed and are observed between the thicker fibers. Consequently, at higher concentrations (25–30 wt.%), high surface tension in porcine gelatin solutions induces local jet instability and jet fragmentation during whipping, causing the unexpected formation of thinner fibers rather than thicker ones due to non-uniform stretching or localized jet collapse. In contrast, fish gelatin solutions exhibit relatively lower viscosity and surface tension, especially at concentrations of 15–20 wt.%, which closely match optimal electrospinning conditions. Lower viscosity allows more effective jet elongation, producing thinner and more uniform nanofibers. Similarly, the lower surface tension contributes positively by reducing bead formation and facilitating fiber thinning. In addition, fish gelatin solutions showed relatively higher electrical conductivity compared to bovine and porcine gelatin at corresponding concentrations. For example, at 25 wt.%, the conductivity of fish gelatin (1780 μS/cm) exceeded that of bovine (1689 μS/cm) and porcine gelatin (1461 μS/cm). This higher conductivity enhanced the electrostatic stretching force on the jet under an applied electric field, further facilitating fiber thinning and elongation. Fish gelatin typically has a lower molecular weight, resulting in an optimal degree of polymer chain entanglement that provides sufficient viscosity for stable jet elongation [38]. As a result of the combined effects of low viscosity and surface tension, along with elevated electrical conductivity, the electrospun nanofibers from fish gelatin in this study exhibited a generally cylindrical morphology, with fiber diameters approximately 1/2–1/3 of those observed for bovine and porcine gelatin fibers. High electrical conductivity increases the jet elongation rate during the whipping stage, providing favorable conditions for the jet to become sufficiently thinned before solidification. As a result, fish gelatin, in addition to its low viscosity and surface tension, also exhibits advantageous electrospinning characteristics in terms of electrical conductivity, contributing to the formation of uniform, cylindrical nanofibers. Conversely, when the electrical conductivity is too low, the electric field may not induce sufficient jet stretching, leading to bead formation or the production of non-uniform fibers. On the other hand, excessively high conductivity can destabilize the jet due to excessive electrostatic forces, potentially resulting in spraying behavior or jet collapse. Therefore, it is essential to maintain an optimal balance among conductivity, viscosity, and surface tension, depending on the type of gelatin used. These findings suggest that electrical conductivity does not solely determine fiber diameter, but rather acts in combination with viscosity and surface tension to influence fiber morphology and structural uniformity. Consequently, fish gelatin consistently produced thin and uniform nanofibers across all tested concentrations, highlighting the favorable molecular characteristics of fish gelatin for electrospinning. In summary, differences in nanofiber morphology and diameter among bovine, porcine, and fish gelatin are influenced by molecular characteristics, including molecular weight and amino acid composition. High viscosity and surface tension of bovine and porcine gelatin solutions, stemming from their relatively higher molecular weights and specific amino acid profiles, restrict effective jet stretching, resulting in partially collapsed or fused irregular fiber structures at higher concentrations. In contrast, the optimal molecular properties of fish gelatin facilitate uniform, thin nanofiber formation, emphasizing the critical role of molecular characteristics in the electrospinning process.

**Figure 2:**
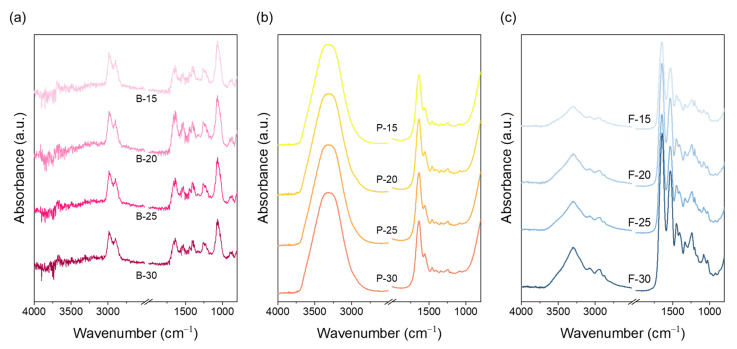
FT-IR spectra of electrospun gelatin nanofibrous webs from (**a**) bovine, (**b**) porcine, and (**c**) fish gelatin at concentrations of 15, 20, 25, and 30 wt.%.

**Figure 3:**
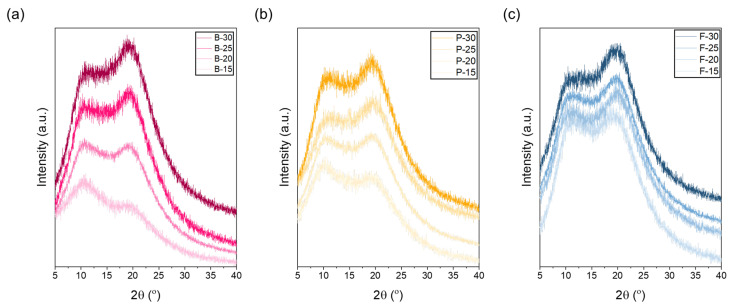
XRD patterns of electrospun gelatin nanofibrous webs from (**a**) bovine, (**b**) porcine, and (**c**) fish gelatin at concentrations of 15, 20, 25, and 30 wt.%. The spectra are vertically shifted for clarity; the relative positions along the vertical axis do not represent absolute values.

The chemical characteristics of electrospun nanofibrous mats prepared from bovine, porcine, and fish gelatin at concentrations of 15, 20, 25, and 30 wt.% were analyzed through FT-IR spectroscopy (Figure 2). Gelatin is a natural polymer derived from the hydrolysis of collagen, which originally retains a triple-helix structure; however, this structure becomes partially disrupted during hydrolysis and further disordered during electrospinning processes [39,40]. During hydrolysis, hydrogen bonds, peptide bonds, and covalent bonds within tropocollagen are cleaved, generating free α-, β-, and γ-chains [38]. Gelatin chains are characterized by repeating Gly-X-Y sequences, where proline frequently occupies the X position and hydroxyproline the Y position [41]. The relative abundance of proline and hydroxyproline is a critical factor determining gelatin’s structural stability, gel strength, and hydrogen-bonding capacity [31]. FT-IR analysis revealed distinct spectral features among the three gelatin types. Bovine gelatin exhibited a prominent C–H stretching peak near 2850 cm^−1^ (amide B), indicating the presence of hydrophobic alkyl groups such as glycine, proline, and hydroxyproline. A relatively weak and narrow O–H/N–H stretching band in the 3300–3100 cm^−1^ range (amide A) was also observed, suggesting tightly packed chains due to hydrophobic interactions. These features reflect the higher proline/hydroxyproline content in bovine gelatin and its greater potential to maintain a residual helical structure during electrospinning. Porcine gelatin showed a slightly weaker C–H peak than bovine gelatin and exhibited the broadest and most intense amide A band among the three samples, indicating reduced alkyl-group content and increased exposure of hydrophilic functional groups. These characteristics enhance water affinity but reduce intramolecular cohesion, thereby lowering structural stability compared to bovine gelatin. Fish gelatin displayed the weakest amide B peak and an amide A band that was narrower and less intense than that of porcine gelatin. This is consistent with its lower proline and hydroxyproline content and lower molecular weight, both of which reduce hydrogen-bonding capacity and hydrophobic packing. These molecular characteristics increase chain flexibility and hydration, resulting in less structural retention after electrospinning and facilitating uniform jet elongation. In addition, fish gelatin showed relatively stronger amide I (~1650 cm^−1^) and amide II (~1500 cm^−1^) bands, reflecting increased random coil content and disordered conformations. Bovine gelatin, on the other hand, showed a more distinct amide III band, suggesting the presence of retained or reassembled secondary structures. Overall, the FT-IR results reflect that the molecular interactions and secondary structure retention among gelatin types are strongly influenced by proline and hydroxyproline content. These interactions are expected to directly impact crystallinity development and molecular rearrangement under electrospinning conditions, which are further examined in the following XRD analysis (Figure 3).

For bovine gelatin (Figure 3a), at a low concentration of 15 wt.%, the intensity of the peak at 11° was higher than that at 21°, indicating that the amorphous structure was relatively dominant. At lower concentrations, abundant free water surrounding gelatin molecules increases the spacing between molecular chains, thereby enhancing molecular mobility and limiting crystallization [42,43,44]. Thus, higher hydration levels weaken intermolecular interactions, leading to an increase in amorphous regions. From the perspective of the electrospinning process, both bovine and porcine gelatins have relatively high molecular weights, which restrict effective jet stretching even at low concentrations (e.g., 15 wt.%). Insufficient stretching during electrospinning can lead to incomplete disruption of gelatin’s triple-helix structure, leaving it partially intact [45,46]. Therefore, the observation that the 11° peak is stronger than the 21° peak at low concentrations could be attributed to incomplete structural deformation during electrospinning, resulting in partial retention of the original triple-helix structure. Typically, electrospinning causes deformation at junctions maintaining the triple helix, thus reducing overall crystallinity. However, if stretching is insufficient, structural changes become limited, preserving part of the initial crystalline structure [47,48]. At concentrations around 20 wt.%, the peak intensities at 11° and 21° were nearly equivalent, representing a critical transition point from amorphous to semi-crystalline structure [49,50]. At this concentration, intermolecular hydrogen bonding and electrostatic interactions begin to strengthen sufficiently to partially overcome the stretching resistance associated with high molecular weight. At concentrations exceeding 25 wt.%, further enhanced intermolecular interactions, such as hydrogen bonding, surpass the stretching resistance caused by high molecular weight. Consequently, molecular chains pack more densely, reducing intermolecular spacing and facilitating the rearrangement of triple-helix structures, thereby significantly increasing overall crystallinity. This structural rearrangement is clearly evidenced by the significantly enhanced intensity of the 21° peak. Porcine gelatin exhibits structural characteristics very similar to those of bovine gelatin across various concentrations. Like bovine gelatin, porcine gelatin has high contents of amino acids such as proline and hydroxyproline, contributing to the stability of its triple-helix structure. This stability clearly appears in the XRD analysis as distinctive peaks at approximately 11° (triple-helix crystalline region) and 21° (amorphous region). At the low concentration of 15 wt.%, the intensity of the 11° peak is higher than that of the 21° peak. This dominance of amorphous structure occurs because abundant free water around gelatin molecules increases intermolecular spacing, thereby increasing molecular mobility, inhibiting crystallization, and resulting in a predominantly amorphous structure [51]. In contrast, fish gelatin has a considerably lower molecular weight and a lower total amino acid content of approximately 17%, significantly lower than bovine and porcine gelatin. Consequently, the formation and stability of the triple-helix structure are limited, as reflected by a generally weak intensity at 11° in XRD analysis. Conversely, peaks near 21°, corresponding to random coil and semi-crystalline structures, appear prominently across all concentrations. Due to its lower molecular weight, fish gelatin experiences less entanglement between molecular chains, facilitating easier jet stretching during electrospinning [52]. Therefore, unlike bovine and porcine gelatin, increasing concentrations of fish gelatin do not significantly enhance crystallinity but predominantly result in increased molecular packing density within amorphous domains. Despite increased molecular packing density at higher concentrations, the inherent low molecular weight and limited proline/hydroxyproline content of fish gelatin restrict effective triple-helix formation result in weak intermolecular bonding and poor water stability. Thus, even increased packing density cannot significantly enhance aqueous stability, maintaining rapid dissolution behavior. This clearly demonstrates that the molecular weight and amino acid composition of fish gelatin are less suitable for stable triple-helix formation.

**Figure 4:**
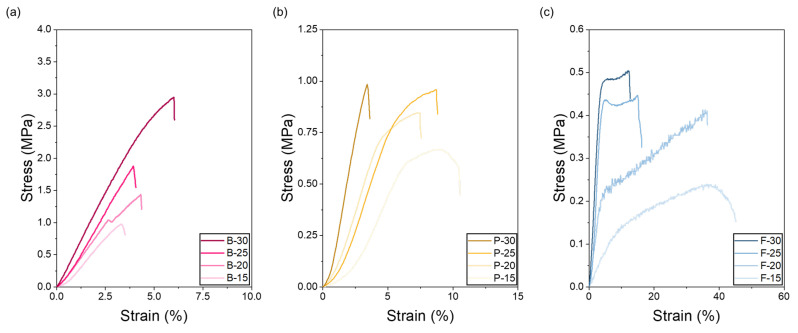
Tensile stress and strain of electrospun gelatin nanofibrous webs from (**a**) bovine, (**b**) porcine, and (**c**) fish gelatin at concentrations of 15, 20, 25, and 30 wt.%.

The mechanical properties of bovine, porcine, and fish gelatin at varying concentrations reflect their unique molecular characteristics and concentration-dependent behaviors (Figure 4). Bovine gelatin exhibited excellent tensile strength properties. Specifically, bovine gelatin at a concentration of 30 wt.% showed a notably high maximum tensile stress of approximately 2.9 MPa, and even at a lower concentration (15 wt.%), it exhibited a relatively high stress of about 1.0 MPa. Such high tensile strength can be attributed to its high content of proline and hydroxyproline, which enhances the stability of the triple-helix structure, and also to its high molecular weight and robust intermolecular hydrogen bonding network [53,54]. High-concentration bovine gelatin demonstrated significant initial tensile stress, rapidly increasing with strain, thus continuously maintaining high mechanical strength. This characteristic can be interpreted as a result of enhanced structural rigidity due to increased intermolecular cohesion and densely packed molecular-chain networks. Porcine gelatin exhibited balanced stress and strain characteristics. At a concentration of 30 wt.%, porcine gelatin demonstrated a maximum tensile stress of approximately 1.0 MPa, while at 15 wt.%, the stress was around 0.7 MPa. In the initial strain region, stress increased gradually; however, beyond a certain strain threshold, tensile stress sharply increased [53]. Although porcine gelatin has molecular characteristics similar to bovine gelatin, its relatively higher molecular weight and viscosity result in greater chain entanglement, allowing simultaneous expression of elasticity and strength [55]. These properties suggest that porcine gelatin is suitable as a versatile material for various applications requiring both flexibility and mechanical strength. Fish gelatin showed particularly notable tensile strain characteristics. At a concentration of 30 wt.%, the maximum tensile stress was approximately 0.5 MPa, and at 15 wt.%, it was around 0.2 MPa. Fish gelatin has a lower molecular weight and lower content of amino acids (proline and hydroxyproline), resulting in fewer molecular-chain entanglements and facilitating better molecular alignment during jet stretching. The tensile stress rapidly increased within the initial strain range but stabilized or gradually decreased beyond a certain strain level. These characteristics indicate that fish gelatin provides excellent flexibility and elasticity, making it especially suitable for applications that require significant elastic deformation [56]. In particular, its combination of moderate tensile strength and high deformability makes it well-suited for biomedical applications such as flexible wound dressings, buccal or transdermal drug delivery films, soft-tissue regeneration scaffolds, and bioadhesive patches that must accommodate continuous movement while maintaining mechanical integrity [57,58,59]. Overall, the tensile stress significantly increased for all gelatin types as concentration increased, attributed to the enhanced intermolecular interactions and higher molecular-chain entanglement density with higher concentration. Bovine gelatin, known for its high mechanical strength and stability in moist environments, is particularly suitable for structural biomedical applications that require long-term integrity. Some studies have demonstrated its effectiveness in bone tissue scaffolds, resorbable implants, and load-bearing wound supports, owing to its dense triple-helix network and post-process crosslinking potential [60,61]. In contrast, fish gelatin exhibits superior elasticity, flexibility, and rapid water solubility, making it ideal for soft-tissue engineering, flexible drug delivery patches, and bioadhesive wound dressings. Its lower molecular weight and reduced proline/hydroxyproline content contribute to its thermo-responsiveness and faster degradation, which are advantageous for temperature-sensitive applications or mucosal delivery platforms [62].

**Figure 5:**
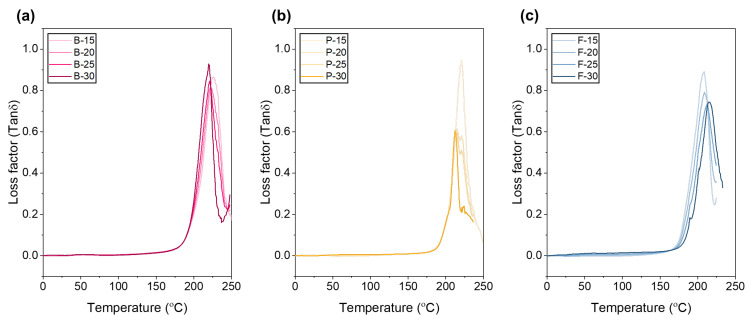
Loss factor (tan δ) as a function of temperature for electrospun gelatin nanofibrous mats from (**a**) bovine, (**b**) porcine, and (**c**) fish gelatin at concentrations of 15, 20, 25, and 30 wt.%.

The peak position and area of the loss factor (tan δ)–temperature graph indicate the relative ratio between viscous energy loss and elastic energy storage within a material, varying with gelatin type and concentration (Figure 5). For bovine gelatin, the tan δ peak temperatures shifted toward lower temperatures with increasing concentration in the following order: B-15 (15 wt.%, 226.9 °C) > B-20 (20 wt.%, 223.7 °C) > B-25 (25 wt.%, 220.5 °C) > B-30 (30 wt.%, 219.5 °C). The peak height and area followed the trend B-15 > B-30 ≥ B-25 > B-20, indicating that at 15 wt.%, bovine gelatin exhibits the highest viscous energy loss and superior vibration damping properties. Conversely, at concentrations of 20–30 wt.%, enhanced structural stability and stronger intermolecular interactions result in more elastic and thermally stable behavior. Porcine gelatin similarly exhibited a decreasing peak temperature with increasing concentration: P-15 (15 wt.%, 220.8 °C) > P-20 (20 wt.%, 214.4 °C) > P-25 (25 wt.%, 213.4 °C) > P-30 (30 wt.%, 212.2 °C). The peak height and area consistently decreased in the same order, reflecting stronger intermolecular interactions and increased elasticity with higher concentrations. Specifically, at the lower concentration of 15 wt.%, increased hydration and wider molecular-chain spacing resulted in maximum peak height and area due to significant internal friction related to alpha-relaxation processes [63]. At higher concentrations (20–30 wt.%), the increased molecular entanglements, hydrogen bonding, and triple-helix structures cause the increase rate of G′ (storage modulus) to exceed that of G″ (loss modulus), thereby decreasing the tan δ values and peak areas, consistent with previous findings. According to Ross-Murphy’s gelation model, gelatin shows the lowest tan δ value around a concentration of approximately 20 ± 2 wt.%, where chain entanglement is maximized and excessive water is removed, leading to dominant elastic behavior [63]. Additionally, fractional-Maxwell fitting indicated that around 20 wt.%, bovine gelatin exhibited the lowest time-dependent loss coefficient and highest cohesive modulus [64]. Consequently, concentrations of 20 wt.% or higher are recommended to maximize mechanical strength and thermal stability, whereas lower concentrations (15 wt.%) are more suitable for applications requiring high damping and viscous energy dissipation. For fish gelatin, peak temperatures increased with concentration in the order F-30 (30 wt.%, 215.0 °C) > F-25 (25 wt.%, 212.4 °C) > F-20 (20 wt.%, 208.6 °C) ≈ F-15 (15 wt.%, 208.6 °C), with peak heights also increasing accordingly. Due to its low molecular weight and low amino acid content, fish gelatin’s limited ability to form strong triple-helix structures and networks with increasing concentration results in increased internal friction and viscous energy loss [65]. However, peak area showed little variation with concentration, suggesting that structural changes and intermolecular interactions remain relatively constant, thereby maintaining consistent viscoelastic characteristics.

**Figure 6:**
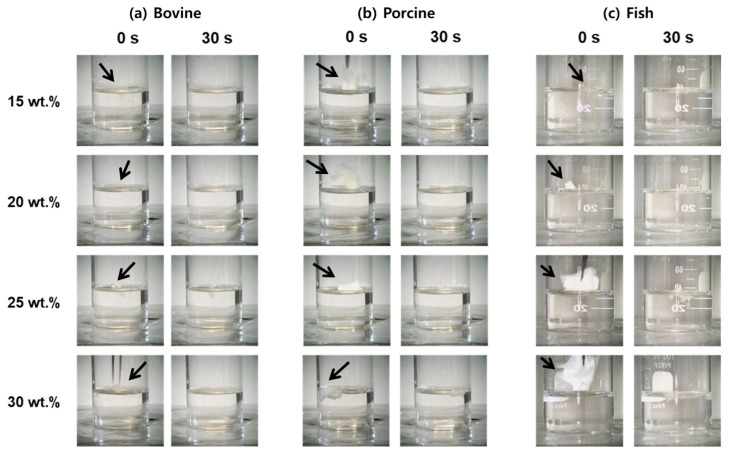
Solubility behavior of electrospun gelatin nanofibrous mats from (**a**) bovine, (**b**) porcine, and (**c**) fish gelatin at concentrations of 15, 20, 25, and 30 wt.% over time (0 and 30 s). The arrow indicates the moment when the nanofiber mat was initially immersed in water.

To assess the aqueous stability of electrospun gelatin nanofibers, underwater solubility was systematically evaluated for bovine, porcine, and fish gelatin mats at concentrations of 15–30 wt.%, with immersion times of 0 and 30 s (Figure 6). For bovine gelatin nanofibers, the lowest concentration (15 wt.%) exhibited immediate transparency upon immersion and was completely dissolved by 30 s, indicating rapid disintegration due to high water uptake and increased chain spacing, which facilitated fast water infiltration [66,67]. At higher concentrations (20–30 wt.%), the nanofibers showed significantly improved structural retention at 30 s, particularly at 30 wt.%, where morphology was clearly preserved. This enhanced water resistance is attributed to increased molecular-chain entanglement and triple-helix stabilization through dense hydrogen bonding. Porcine gelatin nanofibers displayed a similar trend. At 15 wt.%, full dissolution occurred by 30 s. However, at intermediate-to-high concentrations (20–30 wt.%), partial-to-substantial structural integrity was retained after 30 s, with 30 wt.% samples exhibiting the highest water resistance due to their higher molecular weight and strong intermolecular cohesion [32,66]. In contrast, fish gelatin nanofibers showed the fastest dissolution behavior. At low concentrations (15–20 wt.%), fibers completely dissolved by 30 s. Even at 30 wt.%, significant fiber loss and deformation were observed, indicating limited structural stability. This rapid disintegration is attributed to the gelatin’s inherently low molecular weight and reduced proline/hydroxyproline content, which restrict triple-helix formation and hydrogen-bonding capacity [68]. These findings clearly demonstrate that the dissolution rate and aqueous morphological stability of electrospun gelatin nanofibers are strongly dependent on both gelatin type and concentration, offering tunable material designs for application-specific solubility requirements.

These results demonstrate that the solubility and dissolution behavior of gelatin nanofibrous mats can be precisely controlled by adjusting gelatin type and concentration, enabling tailored material design optimized for specific applications. Specifically, fish gelatin nanofibers at low concentrations (15–20 wt.%) are ideal for rapid-release applications such as fast-dissolving drug delivery patches and oral films due to their rapid solubility [69]. This rapid dissolution behavior, combined with fish gelatin’s excellent biocompatibility and low immunogenicity, makes it well-suited for mucosal drug delivery and transbuccal patches, where safe degradation and fast action are critical [70]. Porcine gelatin nanofibers at intermediate concentrations (around 20 wt.%) exhibit moderate solubility characteristics and serve as transient support matrices, making them suitable for cell sheets, wound dressings, or bioactive scaffolds that require short-term structural integrity before degradation [71]. In addition, porcine gelatin has been shown to promote cell adhesion and proliferation, which is beneficial for wound healing applications [19]. High-concentration bovine gelatin nanofibers (30 wt.%) demonstrate superior water resistance and structural retention, enabling their application as long-term scaffolds in tissue engineering and regenerative medicine, particularly in moist or physiological environments. Bovine gelatin scaffolds are known to support osteoblast and fibroblast attachment, and, when properly crosslinked or densified, exhibit low cytotoxicity and high cell compatibility [72]. Such tailored designs are expected to significantly enhance material applicability and performance by precisely meeting the functional requirements of diverse biomedical applications.

## 4. Conclusions

This study systematically investigated the electrospinning conditions, nanofiber morphology, mechanical properties, thermal stability, and solubility behaviors of bovine, porcine, and fish gelatin solutions at concentrations of 15, 20, 25, and 30 wt.%. Optimal viscosity (300–700 mPa·s) facilitated uniform nanofiber formation, with viscosity and surface tension significantly influencing fiber morphology. Notably, fish gelatin consistently produced thinner, more uniform fibers (120–257 nm) across all concentrations tested, whereas bovine gelatin fiber diameters ranged from 521 nm at lower concentrations to 1058 nm at higher concentrations, and porcine gelatin fibers similarly ranged from 712 nm to 1046 nm, due to higher molecular weights and restricted jet stretching. XRD analysis demonstrated a clear concentration-dependent transition from amorphous to crystalline structures for bovine and porcine gelatin above 20 wt.%, driven by enhanced hydrogen bonding and triple-helix rearrangement. Conversely, fish gelatin showed minimal crystallinity changes, attributable to its lower molecular weight and limited triple-helix formation capability. Mechanical testing revealed superior tensile strength in bovine gelatin (2.9 MPa at 30 wt.%), balanced strength and elasticity in porcine gelatin (~1.0 MPa at 30 wt.%), and exceptional elasticity but lower strength in fish gelatin (0.5 MPa at 30 wt.%). DMA analysis indicated that bovine and porcine gelatin exhibited enhanced thermal stability and greater elastic energy storage at increased concentrations (20–30 wt.%), while fish gelatin consistently showed higher damping capacity, indicative of pronounced viscous behavior, irrespective of concentration. Solubility tests confirmed rapid dissolution for low-concentration fish gelatin fibers (within 10 s), moderate and partial dissolution with some structural deformation for intermediate-concentration porcine fibers (20 wt.%), and minimal dissolution with excellent water resistance and structural retention for high-concentration bovine gelatin fibers (30 wt.%). While formal statistical analysis was not performed, consistent and concentration-dependent differences were observed across all measured properties, suggesting clear structure–property correlations based on gelatin origin. These findings enable precise control over gelatin nanofiber properties, providing crucial guidelines for designing tailored gelatin-based nanofiber materials optimized for specific biomedical applications, including rapid-release drug delivery (fish gelatin, 15–20 wt.%), wound dressings (porcine gelatin, 20 wt.%), and stable tissue-engineering scaffolds (bovine gelatin, 30 wt.%).

## Figures and Tables

**Table 1:** Average diameter, viscosity, surface tension, and conductivity for 3 types of gelatins depending on the various concentrations (average diameter values are presented as mean ± standard deviation, *n* = 50). Viscosity, surface tension, and conductivity values are presented as mean ± standard deviation, *n* = 5.

Type of Gelatin	Average Diameter (nm)	Viscosity (mPa·s)	Surface Tension (N/m)	Conductivity (μS/cm)
Bovine 15 wt.% (B-15)	1058 ± 236	1400 ± 41	48.0 ± 1.4	1654 ± 34
Bovine 20 wt.% (B-20)	521 ± 261	2800 ± 81	58.8 ± 1.7	1672 ± 48
Bovine 25 wt.% (B-25)	596 ± 439	4300 ± 110	68.7 ± 2.4	1689 ± 39
Bovine 30 wt.% (B-30)	574 ± 352	7400 ± 220	77.8 ± 2.3	1714 ± 40
Porcine 15 wt.% (P-15)	990 ± 466	2000 ± 57	49.2 ± 1.6	1253 ± 34
Porcine 20 wt.% (P-20)	1046 ± 510	4400 ± 130	75.1 ± 2.4	1357 ± 32
Porcine 25 wt.% (P-25)	746 ± 431	9526 ± 280	105.1 ± 3.4	1461 ± 42
Porcine 30 wt.% (P-30)	712 ± 464	22,126 ± 660	161.7 ± 4.4	1565 ± 33
Fish 15 wt.% (F-15)	120 ± 118	400 ± 11	41.2 ± 1.2	1314 ± 33
Fish 20 wt.% (F-20)	146 ± 141	800 ± 23	49.8 ± 1.5	1547 ± 37
Fish 25 wt.% (F-25)	218 ± 172	18,308 ± 550	64.1 ± 1.9	1780 ± 50
Fish 30 wt.% (F-30)	257 ± 216	23,552 ± 710	73.0 ± 2.2	2013 ± 43

## Data Availability

The data that support the findings of this study are available within the article.

## References

[1] Xue J., Wu T., Dai Y., Xia Y. (2019). Electrospinning and Electrospun Nanofibers: Methods, Materials, and Applications. Chem. Rev..

[2] Haider A., Haider S., Kang I.-K. (2018). A Comprehensive Review Summarizing the Effect of Electrospinning Parameters and Potential Applications of Nanofibers in Biomedical and Biotechnology. Arab. J. Chem..

[3] Lu Y., Huang J., Yu G., Cardenas R., Wei S., Wujcik E.K., Guo Z. (2016). Coaxial Electrospun Fibers: Applications in Drug Delivery and Tissue Engineering. Wiley Interdiscip. Rev. Nanomed. Nanobiotechnol..

[4] Pezeshki-Modaress M., Mirzadeh H., Zandi M. (2015). Gelatin–Gag Electrospun Nanofibrous Scaffold for Skin Tissue Engineering: Fabrication and Modeling of Process Parameters. Mater. Sci. Eng. C.

[5] Nezarati R.M., Eifert M.B., Cosgriff-Hernandez E. (2013). Effects of Humidity and Solution Viscosity on Electrospun Fiber Morphology. Tissue Eng. Part C Methods.

[6] Leach M.K., Feng Z.Q., Tuck S.J., Corey J.M. (2011). Electrospinning Fundamentals: Optimizing Solution and Apparatus Parameters. J. Vis. Exp..

[7] Garg K., Bowlin G.L. (2011). Electrospinning Jets and Nanofibrous Structures. Biomicrofluidics.

[8] Liu Z., Ramakrishna S., Ahmed I., Rudd C., Liu X. (2022). Rheological, Surface Tension and Conductivity Insights on the Electrospinnability of Poly(Lactic-Co-Glycolic Acid)-Hyaluronic Acid Solutions and Their Correlations with the Nanofiber Morphological Characteristics. Polymers.

[9] Ksapabutr B., Chalermkiti T., Panapoy M. (2005). Effect of Nozzle Shapes on the Formation of Taylor Cone and the Oscillation of Fibers during Electrospinning Process. Chiang Mai Univ. J..

[10] Xu L., Lv J., Wang X., Qu W. (2023). Wave Propagation of Bending Jet in Electrospinning Process. AIP Adv..

[11] Ewaldz E., Randrup J., Brettmann B. (2022). Solvent Effects on the Elasticity of Electrospinnable Polymer Solutions. ACS Polym. Au.

[12] Zheng Y., Xin B., Li M. (2019). Model Development and Validation of Electrospun Jet Formation. Text. Res. J..

[13] Morad M.R., Rajabi A., Razavi M., Sereshkeh S.R.P. (2016). A Very Stable High Throughput Taylor Cone-Jet in Electrohydrodynamics. Sci. Rep..

[14] Li X., Li Z., Wang L., Ma G., Meng F., Pritchard R.H., Gill E.L., Liu Y., Huang Y.Y.S. (2016). Low-Voltage Continuous Electrospinning Patterning. ACS Appl. Mater. Interfaces.

[15] Reneker D.H., Yarin A.L., Fong H., Koombhongse S. (2000). Bending Instability of Electrically Charged Liquid Jets of Polymer Solutions in Electrospinning. J. Appl. Phys..

[16] Gómez-Guillén M., Giménez B., López-Caballero M.a., Montero M. (2011). Functional and Bioactive Properties of Collagen and Gelatin from Alternative Sources: A Review. Food Hydrocoll..

[17] Echave M.C., Hernáez-Moya R., Iturriaga L., Pedraz J.L., Lakshminarayanan R., Dolatshahi-Pirouz A., Taebnia N., Orive G. (2019). Recent Advances in Gelatin-Based Therapeutics. Expert Opin. Biol. Ther..

[18] Yao C.-H., Lee C.-Y., Huang C.-H., Chen Y.-S., Chen K.-Y. (2017). Novel Bilayer Wound Dressing Based on Electrospun Gelatin/Keratin Nanofibrous Mats for Skin Wound Repair. Mater. Sci. Eng. C.

[19] Aldana A.A., Abraham G.A. (2017). Current Advances in Electrospun Gelatin-Based Scaffolds for Tissue Engineering Applications. Int. J. Pharm..

[20] Wu G., Bazer F.W., Burghardt R.C., Johnson G.A., Kim S.W., Knabe D.A., Li P., Li X., McKnight J.R., Satterfield M.C. (2011). Proline and Hydroxyproline Metabolism: Implications for Animal and Human Nutrition. Amino Acids.

[21] Umumararungu T., Gahamanyi N., Mukiza J., Habarurema G., Katandula J., Rugamba A., Kagisha V. (2024). Proline, a Unique Amino Acid Whose Polymer, Polyproline Ii Helix, and Its Analogues Are Involved in Many Biological Processes: A Review. Amino Acids.

[22] Hall D.A., Reed R. (1957). Hydroxyproline and Thermal Stability of Collagen. Nature.

[23] Saenmuang S., Phothiset S., Chumnanka C. (2020). Extraction and Characterization of Gelatin from Black-Bone Chicken by-Products. Food Sci. Biotechnol..

[24] Said N.S., Sarbon N.M. (2022). Physical and Mechanical Characteristics of Gelatin-Based Films as a Potential Food Packaging Material: A Review. Membranes.

[25] Karim A.A., Bhat R. (2009). Fish Gelatin: Properties, Challenges, and Prospects as an Alternative to Mammalian Gelatins. Food Hydrocoll..

[26] Baziwane D., He Q. (2003). Gelatin: The Paramount Food Additive. Food Rev. Int..

[27] Reneker D.H., Yarin A.L. (2008). Electrospinning Jets and Polymer Nanofibers. Polymer.

[28] Zeng J., Xu X., Chen X., Liang Q., Bian X., Yang L., Jing X. (2003). Biodegradable Electrospun Fibers for Drug Delivery. J. Control. Release.

[29] Xiang L., Cui W. (2021). Biomedical Application of Photo-Crosslinked Gelatin Hydrogels. J. Leather Sci. Eng..

[30] Derkach S.R., Kolotova D.S., Kuchina Y.A., Shumskaya N.V. (2022). Characterization of Fish Gelatin Obtained from Atlantic Cod Skin Using Enzymatic Treatment. Polymers.

[31] Hafidz R., Yaakob C., Amin I., Noorfaizan A. (2011). Chemical and Functional Properties of Bovine and Porcine Skin Gelatin. Int. Food Res. J..

[32] Niehues E., Quadri M. (2017). Spinnability, Morphology and Mechanical Properties of Gelatins with Different Bloom Index. Braz. J. Chem. Eng..

[33] Yeum J.H., Yang S.B., Sabina Y., Haider S., Haider A. (2016). Fabrication of Highly Aligned Poly (Vinyl Alcohol) Nanofibers and Its Yarn by Electrospinning. Electrospinning-Material, Techniques, and Biomedical Applications.

[34] Yang S.B., Yeum J.H. (2017). Morphological Comparison of Aligned Poly (Vinyl Alcohol) Nanofibers Fabricated by Modified Electrospinning and Centrifugal Jet Spinning Techniques. J. Nanosci. Nanotechnol..

[35] López-Herrera J., Riesco-Chueca P., Gañán-Calvo A. (2005). Linear Stability Analysis of Axisymmetric Perturbations in Imperfectly Conducting Liquid Jets. Phys. Fluids.

[36] Forgie J.R.P., Leclinche F., Dréan E., Dolez P.I. (2023). Electrospinning of High-Performance Nanofibres: State of the Art and Insights into the Path Forward. Appl. Sci..

[37] Refate A., Mohamed Y., Mohamed M., Sobhy M., Samhy K., Khaled O., Eidaroos K., Batikh H., El-Kashif E., El-Khatib S. (2023). Influence of Electrospinning Parameters on Biopolymers Nanofibers, with Emphasis on Cellulose & Chitosan. Heliyon.

[38] de Farias B.S., Rizzi F.Z., Ribeiro E.S., Diaz P.S., Sant’Anna Cadaval Junior T.R., Dotto G.L., Khan M.R., Manoharadas S., de Almeida Pinto L.A., Dos Reis G.S. (2023). Influence of Gelatin Type on Physicochemical Properties of Electrospun Nanofibers. Sci. Rep..

[39] Akbarzadegan R., Ahari H., Sharifan A., Anvar A.A. (2021). Overview of the Studies on Authentication of Gelatin Using Fourier Transform Infrared Spectroscopy Coupled with Chemometrics. Hum. Health Halal Metr..

[40] Muyonga J.H., Cole C.G.B., Duodu K.G. (2004). Fourier Transform Infrared (Ftir) Spectroscopic Study of Acid Soluble Collagen and Gelatin from Skins and Bones of Young and Adult Nile Perch (Lates Niloticus). Food Chem..

[41] Chen D., Cen K., Zhuang X., Gan Z., Zhou J., Zhang Y., Zhang H. (2022). Insight into Biomass Pyrolysis Mechanism Based on Cellulose, Hemicellulose, and Lignin: Evolution of Volatiles and Kinetics, Elucidation of Reaction Pathways, and Characterization of Gas, Biochar and Bio-Oil. Combust. Flame.

[42] Vaca Chavez F., Hellstrand E., Halle B. (2006). Hydrogen Exchange and Hydration Dynamics in Gelatin Gels. J. Phys. Chem. B.

[43] Kunitz M. (1927). Hydration of Gelatin in Solution. J. Gen. Physiol..

[44] Kéri M., Forgács A., Papp V., Bányai I., Veres P., Len A., Dudás Z., Fábián I., Kalmár J. (2020). Gelatin Content Governs Hydration Induced Structural Changes in Silica-Gelatin Hybrid Aerogels - Implications in Drug Delivery. Acta Biomater..

[45] Wang H., Xia R., Zhou M., Williams G.R., Amler E., Zhou F.L., Tamaddon M., Liu C. (2025). The Development of a Coaxial Electrospinning Formula Using Fish Gelatin/Pbs as the Core for Structurally Intact Liposome Loading and Release. Polymers.

[46] Derkach S.R., Voron’ko N.G., Kuchina Y.A., Kolotova D.S. (2020). Modified Fish Gelatin as an Alternative to Mammalian Gelatin in Modern Food Technologies. Polymers.

[47] Chen X., Meng J., Xu H., Shinoda M., Kishimoto M., Sakurai S., Yamane H. (2021). Fabrication and Properties of Electrospun Collagen Tubular Scaffold Crosslinked by Physical and Chemical Treatments. Polymers.

[48] Steyaert I., Rahier H., Van Vlierberghe S., Olijve J., De Clerck K. (2016). Gelatin Nanofibers: Analysis of Triple Helix Dissociation Temperature and Cold-Water-Solubility. Food Hydrocoll..

[49] Wang Z., Schaller M., Petzold A., Saalwächter K., Thurn-Albrecht T. (2023). How Entanglements Determine the Morphology of Semicrystalline Polymers. Proc. Natl. Acad. Sci. USA.

[50] Fernández-Tena A., Pérez-Camargo R.A., Coulembier O., Sangroniz L., Aranburu N., Guerrica-Echevarria G., Liu G., Wang D., Cavallo D., Müller A.J. (2023). Effect of Molecular Weight on the Crystallization and Melt Memory of Poly(Ε-Caprolactone) (Pcl). Macromolecules.

[51] Mirbaha H., Scardi P., D’Incau M., Arbab S., Nourpanah P., Pugno N.M. (2020). Supramolecular Structure and Mechanical Properties of Wet-Spun Polyacrylonitrile/Carbon Nanotube Composite Fibers Influenced by Stretching Forces. Front. Mater..

[52] Lin T., Li Y., Wang J., You J. (2021). Effect of Pmma Molecular Weight on Its Localization during Crystallization of Pvdf in Their Blends. Polymers.

[53] Mosleh Y., de Zeeuw W., Nijemeisland M., Bijleveld J.C., van Duin P., Poulis J.A. (2021). The Structure–Property Correlations in Dry Gelatin Adhesive Films. Adv. Eng. Mater..

[54] Mad-Ali S., Benjakul S., Prodpran T., Maqsood S. (2015). Characteristics and Gel Properties of Gelatin from Goat Skin as Influenced by Alkaline-Pretreatment Conditions. Asian-Australas. J. Anim. Sci..

[55] Butcher A., Oyen M. (2016). Mechanical Properties of Electrospun Gelatin Scaffolds. Front. Bioeng. Biotechnol..

[56] Suhaima N.R., Suyatma N.E., Hunaefi D., Jayanegara A. (2022). Comparison of Fish and Mammalian Gelatin Film Properties: A Meta-Analysis. AIMS Agric. Food.

[57] Li M., Xia W., Khoong Y.M., Huang L., Huang X., Liang H., Zhao Y., Mao J., Yu H., Zan T. (2023). Smart and Versatile Biomaterials for Cutaneous Wound Healing. Biomater. Res..

[58] de Carvalho A.C.W., Paiva N.F., Demonari I.K., Duarte M.P.F., do Couto R.O., de Freitas O., Vicentini F. (2023). The Potential of Films as Transmucosal Drug Delivery Systems. Pharmaceutics.

[59] Prausnitz M.R., Langer R. (2008). Transdermal Drug Delivery. Nat. Biotechnol..

[60] Echave M., Erezuma I., Golafshan N., Castilho M., Kadumudi F., Pimenta-Lopes C., Ventura F., Pujol A., Jimenez J., Camara J. (2022). Bioinspired Gelatin/Bioceramic Composites Loaded with Bone Morphogenetic Protein-2 (Bmp-2) Promote Osteoporotic Bone Repair. Biomater. Adv..

[61] Huang Y., Chen T., Ren C., Bao B., Huang R., Sun Y., Yu C., Yang Y., Wong W.T., Zeng Q. (2025). High-Strength Gelatin Hydrogel Scaffold with Drug Loading Remodels the Inflammatory Microenvironment to Enhance Osteoporotic Bone Repair. Adv. Mater..

[62] Mohite P., Puri A., Munde S., Dave R., Khan S., Patil R., Singh A.K., Tipduangta P., Singh S., Chittasupho C. (2025). Potential of Chitosan/Gelatin-Based Nanofibers in Delivering Drugs for the Management of Varied Complications: A Review. Polymers.

[63] Michon C., Cuvelier G., Launay B., Parker A. (1996). Concentration Dependence of the Properties of Gelatin and Iota-Carrageenan Systems at the Gel Point. J. De Chim. Phys..

[64] Moučka R., Sedlačík M., Pátíková Z. (2023). Fractional Viscoelastic Models of Porcine Skin and Its Gelatin-Based Surrogates. Mech. Mater..

[65] Karydis-Messinis A., Moschovas D., Markou M., Tsirka K., Gioti C., Bagli E., Murphy C., Giannakas A.E., Paipetis A., Karakassides M.A. (2023). Hydrogel Membranes from Chitosan-Fish Gelatin-Glycerol for Biomedical Applications: Chondroitin Sulfate Incorporation Effect in Membrane Properties. Gels.

[66] Ghorani B., Emadzadeh B., Rezaeinia H., Russell S.J. (2020). Improvements in Gelatin Cold Water Solubility after Electrospinning and Associated Physicochemical, Functional and Rheological Properties. Food Hydrocoll..

[67] Erencia M., Cano F., Tornero J.A., Fernandes M.M., Tzanov T., Macanás J., Carrillo F. (2015). Electrospinning of Gelatin Fibers Using Solutions with Low Acetic Acid Concentration: Effect of Solvent Composition on Both Diameter of Electrospun Fibers and Cytotoxicity. J. Appl. Polym. Sci..

[68] Deng L., Li Y., Feng F., Zhang H. (2019). Study on Wettability, Mechanical Property and Biocompatibility of Electrospun Gelatin/Zein Nanofibers Cross-Linked by Glucose. Food Hydrocoll..

[69] Tammineni N., Rasco B., Powers J., Nindo C., Ünlü G. (2014). Bovine and Fish Gelatin Coatings Incorporating Tannins: Effect on Physical Properties and Oxidative Stability of Salmon Fillets. J. Food Chem. Nutr..

[70] Azizah F., Nursakti H., Ningrum A., Supriyadi (2023). Development of Edible Composite Film from Fish Gelatin-Pectin Incorporated with Lemongrass Essential Oil and Its Application in Chicken Meat. Polymers.

[71] Michelini L., Probo L., Farè S., Negrini N.C. (2020). Characterization of Gelatin Hydrogels Derived from Different Animal Sources. Mater. Lett..

[72] Bigi A., Cojazzi G., Panzavolta S., Rubini K., Roveri N. (2001). Mechanical and Thermal Properties of Gelatin Films at Different Degrees of Glutaraldehyde Crosslinking. Biomaterials.

